# Nuclear Factor-κB Dysregulation and α-Synuclein Pathology: Critical Interplay in the Pathogenesis of Parkinson’s Disease

**DOI:** 10.3389/fnagi.2020.00068

**Published:** 2020-03-24

**Authors:** Arianna Bellucci, Luigi Bubacco, Francesca Longhena, Edoardo Parrella, Gaia Faustini, Vanessa Porrini, Federica Bono, Cristina Missale, Marina Pizzi

**Affiliations:** ^1^Division of Pharmacology, Department of Molecular and Translational Medicine, University of Brescia, Brescia, Italy; ^2^Department of Biology, University of Padua, Padua, Italy

**Keywords:** Parkinson’s disease, α-synuclein, nuclear factor-κB (NF-κB), Rel A, c-Rel ko mice

## Abstract

The loss of dopaminergic neurons of the nigrostriatal system underlies the onset of the typical motor symptoms of Parkinson’s disease (PD). Lewy bodies (LB) and Lewy neurites (LN), proteinaceous inclusions mainly composed of insoluble α-synuclein (α-syn) fibrils are key neuropathological hallmarks of the brain of affected patients. Compelling evidence supports that in the early prodromal phases of PD, synaptic terminal and axonal alterations initiate and drive a retrograde degeneration process culminating with the loss of nigral dopaminergic neurons. This notwithstanding, the molecular triggers remain to be fully elucidated. Although it has been shown that α-syn fibrillary aggregation can induce early synaptic and axonal impairment and cause nigrostriatal degeneration, we still ignore how and why α-syn fibrillation begins. Nuclear factor-κB (NF-κB) transcription factors, key regulators of inflammation and apoptosis, are involved in the brain programming of systemic aging as well as in the pathogenesis of several neurodegenerative diseases. The NF-κB family of factors consists of five different subunits (c-Rel, p65/RelA, p50, RelB, and p52), which combine to form transcriptionally active dimers. Different findings point out a role of RelA in PD. Interestingly, the nuclear content of RelA is abnormally increased in nigral dopamine (DA) neurons and glial cells of PD patients. Inhibition of RelA exert neuroprotection against (1-methyl-4-phenyl-1,2,3,6-tetrahydropyridine) MPTP and 1-methyl-4-phenylpyridinium (MPP+) toxicity, suggesting that this factor decreases neuronal resilience. Conversely, the c-Rel subunit can exert neuroprotective actions. We recently described that mice deficient for c-Rel develop a PD-like motor and non-motor phenotype characterized by progressive brain α-syn accumulation and early synaptic changes preceding the frank loss of nigrostriatal neurons. This evidence supports that dysregulations in this transcription factors may be involved in the onset of PD. This review highlights observations supporting a possible interplay between NF-κB dysregulation and α-syn pathology in PD, with the aim to disclose novel potential mechanisms involved in the pathogenesis of this disorder.

## Introduction

The therapeutic management of Parkinson’s disease (PD) patients is one of the major challenges in the field of neurodegenerative diseases nowadays (Orayj and Lane, 2019). This is mostly related to two determinants. (1) Although since many years the administration of levodopa (L-Dopa) has allowed motor symptom recovery, its efficacy has a limited time window and associates with severe adverse effects, thus gathering the unmet need of novel interventions (Olanow and Stocchi, 2018). (2) In spite of more than 200 years of research in the field of PD, we still have poor discernment on the main pathogenic mechanisms. The only certainties we own relate to the neuropathological features of post-mortem PD brains. These present a prominent loss of dopaminergic nigrostriatal neurons and contain profuse insoluble proteinaceous aggregates named Lewy bodies (LB) and Lewy neurites (LN), likely originating from fibrillary α-synuclein (α-syn) protein accumulation in cell bodies and neurites, respectively (Spillantini et al., 1997; Spillantini and Goedert, 2000). This notwithstanding, we ignore the key molecular determinants initiating the degeneration of nigrostriatal neurons and/or α-syn aggregation and deposition. The molecular features of the toxic α-syn species driving neurodegeneration are also an unsolved conundrum (Longhena et al., 2019).

Investigations on experimental models reproducing human α-syn aggregate accumulation, or transgenic expression of mutant forms of the protein associated with familial PD as well as more extensive neuropathological examinations and brain imaging studies in patients, have disclosed that the most probable initiation sites for neuronal degeneration in PD are synapses and axonal projections (for review, please see Bellucci et al., 2017; Uchihara, 2017; Wong et al., 2019). Moreover, the selective neuronal vulnerability of nigral dopaminergic neurons in PD has been proposed to derive from their massive neuronal arborization and peculiar metabolic and functional profile (Surmeier et al., 2017; Wong et al., 2019). In this scenario, α-syn pathological aggregation at synapses appears to play a major role in triggering dopaminergic neuron dysfunction, flowing in a retrograde degeneration pattern involving axonal projections first and culminating with neuronal cell death (Bellucci et al., 2016, 2017; Wong et al., 2019). In particular, the pivotal role exerted by α-syn within dopaminergic neurons terminals as a regulator of neurotransmitter synthesis, reuptake and vesicle storage or motility (Bellucci et al., 2012b; Longhena et al., 2019), hints that this neuronal population may be more vulnerable to minimal perturbations of functional homeostasis induced by α-syn accumulation and aggregation. Moreover, the massive energy consumption rate of dopaminergic neurons has to be combined with an enhanced mitochondrial bioenergetics support. This is lost upon synapse degeneration in the brain of PD patients, although it appears to be upregulated with a compensatory fashion in the remaining terminals (Reeve et al., 2018). On this line, findings indicating that α-syn physiologically regulates mitochondrial homeostasis (Guardia-Laguarta et al., 2014; Faustini et al., 2019), while its aggregation prompts mitochondrial dysfunction (Nakamura et al., 2011; Tapias et al., 2017; Wang et al., 2019), support that early pathological shifts in the conformation of this protein, leading to its loss of function and accumulation, may hinder the bioenergetics profile of dopaminergic neurons, compromising their resilience along aging. This hypothesis offers a bridge between two key biological processes that are thought to participate in PD pathogenesis: mitochondrial alterations and α-syn deposition, though it is still arduous to determine who is on first, as mitochondrial impairment can also trigger α-syn pathological aggregation (Betarbet et al., 2002; Zaltieri et al., 2015; Faustini et al., 2018; Grunewald et al., 2019).

Beside α-syn pathology and mitochondrial impairment, numerous other mechanisms have been proposed to contribute to sporadic PD pathogenesis, including neuroinflammation, impaired autophagy, and oxidative stress (Pang et al., 2019). Interestingly, some of these molecular pathways appear to be cross-linked and can be regulated by nuclear factor κB (NF-κB) transcription factors (Kratsovnik et al., 2005; Djavaheri-Mergny et al., 2007; Sarnico et al., 2009a; Morgan and Liu, 2011; Lanzillotta et al., 2015; Kaminska et al., 2016; Nivon et al., 2016; Lingappan, 2018; Nandy et al., 2018). In particular, oxidative stress can activate NF-κB-mediated protective signaling (Kratsovnik et al., 2005; Lingappan, 2018), which can repress autophagy as well as autophagy-dependent apoptosis (Djavaheri-Mergny et al., 2007; Nandy et al., 2018). On the other hand, NF-κB-regulated genes play a major role in controlling the amount of ROS in the cell (Morgan and Liu, 2011; Lingappan, 2018), and by modulating autophagic activity, this factor appears as a key regulator of protein aggregate clearance (Nivon et al., 2016). Upon exposure of microglia to lipopolysaccharide (LPS), NF-κB is among the predominantly activated signaling pathways and initiates the transcription of proinflammatory gene coding for cytokines and proteolytic enzymes (Kaminska et al., 2016). However, the different composition of NF-κB dimers imprints either the protective or the noxious action of this factor. While p50/RelA dimers induce pro-apoptotic Bim and Noxa genes, c-Rel-containing dimers exert neuroprotective actions (Inta et al., 2006; Sarnico et al., 2009b).

NF-κB factors play a crucial role in the regulation of inflammation and apoptosis, are involved in the brain programming of systemic aging, as well as in the pathogenesis of several neurodegenerative diseases (Sarnico et al., 2009a; Mincheva-Tasheva and Soler, 2013; Lanzillotta et al., 2015). The NF-κB family of transcription factors consists of five different subunits (c-Rel, p65/RelA, p50, RelB, and p52), which interact to form transcriptionally active homo and heterodimers (Perkins, 1997; Chen and Greene, 2004; Ghosh et al., 2012). Different findings point to a role of NF-κB/RelA in PD. Interestingly, the nuclear content of RelA is abnormally increased in nigral dopaminergic neurons and glial cells of PD patients. Inhibition of RelA prevents dopaminergic neuron loss in a 1-methyl-4-phenyl-1,2,3,6-tetrahydropyridine (MPTP)-mouse model of PD, and downregulation of RelA protects neurons from 1-methyl-4-phenylpyridinium (MPP+) toxicity, suggesting that RelA upregulation, may play a role in dopaminergic neuron degeneration (Ghosh et al., 2007). Moreover, we recently described that mice deficient for c-Rel subunit, which can exert pro-survival effects (Pizzi et al., 2002; Sarnico et al., 2009b), develop a PD-like motor and non-motor phenotype characterized by progressive brain α-syn accumulation and early synaptic changes preceding the frank loss of nigrostriatal neurons (Baiguera et al., 2012; Parrella et al., 2019). This evidence hints that a reduction in the protective function of c-Rel may render dopaminergic neurons more vulnerable to aging, the primary risk factor for PD (Collier et al., 2011; Reeve et al., 2014), thus predisposing toward the development of this disorder.

This review provides an updated critical overview of findings supporting a possible interplay between NF-κB dysregulation and α-syn pathology in PD pathogenesis, with the aim to uncover and discuss novel potential molecular mechanisms involved in this process.

## α-Synuclein: Physiological Function and Role in PD

α-synuclein is a neuronal protein mainly localized at synaptic sites. Its physiological functions seem to be mostly related with regulation of neurotransmitter release and recycle, as it modulates the size, assembly, and release of synaptic vesicle pools (Murphy et al., 2000; Burre, 2015; Fusco et al., 2016), neurotransmitter reuptake (Burre, 2015; Longhena et al., 2019), exocytotic fusion pore dilation (Logan et al., 2017), and neurotransmitter vesicular uptake (Guo et al., 2008; Pifl et al., 2014; Phan et al., 2017). Remarkably, the multiplicity of α-syn interactions at synaptic sites, coupled with its intrinsic structural plasticity, can account for the cardinal role of the protein at terminals (Bendor et al., 2013; Longhena et al., 2019; Sulzer and Edwards, 2019). Studies in PD brains and experimental models have shown that α-syn overexpression and aggregation induce significant alterations of synaptic proteins, neuronal dysfunction and degeneration as well as motor deficits (Fleming et al., 2004; Garcia-Reitbock et al., 2010; Bellucci et al., 2011; Lam et al., 2011; Lundblad et al., 2012; Wang et al., 2014; Visanji et al., 2016).

Nonetheless, α-syn has been found to localize in and affect other cellular compartments aside synapses. In the nucleus, the protein physiologically interacts with and regulates histones (Goers et al., 2003; Kontopoulos et al., 2006; Schaser et al., 2019). On the other hand, its overexpression and phosphorylation modulates gene expression (Pinho et al., 2019), impairs the neuroprotective NF-κB signaling pathway (Yuan et al., 2008), and regulates the promoter of proliferator-activated receptor gamma coactivator 1 α (PGC1α), a transcription factor governing mitochondria biogenesis, to inhibit its transcription (Siddiqui et al., 2012).

The overexpression of wild-type (wt) α-syn, and even more of its A53T mutated form, affects endoplasmic reticulum (ER)/Golgi transport by direct binding of the soluble proteins to ER/Golgi soluble *N*-ethylmaleimide-sensitive factor attachment protein receptors (SNAREs), resulting in their inhibition (Thayanidhi et al., 2010). Interestingly, this effect appears to be rescued by Rab1 overexpression and is responsible for lysosomal dysfunction onset (Cooper et al., 2006; Martinez-Vicente et al., 2008; Mazzulli et al., 2016; Hoffmann et al., 2019). Alterations in Golgi morphology and increased susceptibility to ER stress have been also found to occur in dopaminergic cells overexpressing A30P α-syn (Paiva et al., 2018).

In addition, we showed that overexpression of wt α-syn, leading to formation of insoluble aggregates within the ER, activates the protein kinase RNA-like ER kinase (PERK)-related pathway of the unfolded protein response (UPR) (Bellucci et al., 2011). Upon misfolded protein accumulation in the ER, the induced UPR inhibits protein synthesis and generation of molecular chaperones implicated in protein folding, whose activation may ultimately lead to apoptotic cell death. These findings have been further corroborated by other studies supporting that ER stress is relevant for the manifestations of synucleinopathy *in vivo* (Colla et al., 2012a, b; Mercado et al., 2013). For these main reasons, therapeutic strategies targeting the UPR have been proposed as PD treatments (Bellucci et al., 2012a; Mercado et al., 2018; Martinez et al., 2019).

α-synuclein can also bind mitochondria-associated ER membranes (MAM) (Guardia-Laguarta et al., 2014), and evidence indicating that its absence impairs mitochondria lipid composition, function, fusion, and trafficking (Ellis et al., 2005; Faustini et al., 2019) supports that the protein can play a physiological role in the regulation of mitochondrial homeostasis. This hypothesis is in line with findings showing that pathological α-syn aggregates or oligomers produce mitochondria fragmentation, impair mitochondrial trafficking, and lead to respiration failure (Devi et al., 2008; Nakamura et al., 2011; Tapias et al., 2017; Prots et al., 2018; Wang et al., 2019).

Therefore, although synapses, which result the sites where α-syn is most abundant, are likely the first sites to be affected by and suffer from its pathological changes, it is reasonable to prospect that α-syn aggregation and toxicity may proceed progressively through the induction of dysfunctional alterations to other cellular compartments. This view implies that a finer characterization of the pathological changes occurring after the initiation of α-syn aggregation may bring new insight into PD pathogenesis. On the other hand, it reinforces that studies exploring the factors leading or predisposing to α-syn accumulation and aggregation along aging are even more important, as they may help us to identify new targets for the development of therapies halting PD progression.

Another peculiarity of α-syn relates to its transmission from cell-to-cell and to the spreading of its pathological aggregates from the peripheral nervous system (PNS) to the central nervous system (CNS), or vice versa, in experimental models (Luk et al., 2012; Ulusoy et al., 2013, 2017; Recasens and Dehay, 2014; Dehay et al., 2016; Emmanouilidou and Vekrellis, 2016; Helwig et al., 2016; Cavaliere et al., 2017; Rutherford et al., 2017; Grozdanov and Danzer, 2018). The capability of pathological forms of α-syn to spread seems to be corroborated by neuropathological examination of post-mortem brains from patients who received fetal neuron grafts over one decade prior to death and showing the development of LB within grafted neuronal cells (Kordower et al., 2008a, b; Chu and Kordower, 2010; Li et al., 2010; Kurowska et al., 2011). These findings, together with the Braak hypothesis, suggesting that the progression of PD symptoms relates with the caudo-rostral diffusion of LB pathology in the brain (Braak et al., 2003, 2018), fed the shoot up of the prion-like hypothesis of PD, emphasizing the multiple similarities between α-syn and prion protein (Olanow and Brundin, 2013; Brundin et al., 2016; Brundin and Melki, 2017). However, studies showing that PD patients exhibit a systemic α-syn neuropathology within both PNS and CNS (Gelpi et al., 2014; Surguchov, 2016) and a critical analysis of neuronal vulnerability to α-syn accumulation, were supportive for the development of alternative hypotheses as presented by Engelender and Isacson (2017) and Surmeier et al. (2017). Very recent findings showing that systemic delivery of α-syn synthetic pre-formed fibrils in rats trigger pathological transformation of endogenous α-syn, leading to neurodegeneration in discrete CNS and PNS neuronal populations (Kuan et al., 2019), bring novel insights into this subject, opening the way to a more exhaustive comprehension of the role and relevance of α-syn spreading in PD.

## NF-κB Factors: Transcriptional Regulators Governing Neuroinflammation, Apoptosis, Neuronal Function and Resilience

NF-κB is expressed in both the CNS and PNS and localizes in neurons, glial cells, and Schwann cells mostly as p50/p50 homodimers and p50/RelA heterodimers (Meffert and Baltimore, 2005). By regulating synaptic signaling and behavior, or pivotally controlling cell survival and glial cell activation, the NF-κB family of transcription factors is crucially involved in the regulation of CNS and PNS response to physiological and pathological stimuli (Meffert et al., 2003; Meffert and Baltimore, 2005; Mattson and Meffert, 2006; Mincheva-Tasheva and Soler, 2013).

NF-κB factors are ubiquitously expressed in mammalian cells, although they were first identified in lymphocytes (Meffert and Baltimore, 2005). All the NF-κB subunits, highly conserved across species, show a Rel homology domain containing the key functional regions for DNA binding, dimerization, nuclear translocation, and interaction with their inhibitory elements named IκB. Only RelA, c-Rel, and RelB show the C-terminal transactivation domain (TAD) that allows the dimer to initiate transcription (Hayden and Ghosh, 2012). Although p52 and p50 lack TADs, their heterodimerization with TAD-containing NF-κB subunits, or interaction with non-Rel proteins that have trans-activating capability, allows them to positively regulate transcription. The homodimers composed by p50 and p52 can also negatively regulate transcription by competing with TAD-containing dimers for binding to κB sites or by constitutively occupying some κB sites to increase the activation threshold for certain NF-κB target genes (Hayden and Ghosh, 2012).

Inactive NF-κB dimers localize in the cytoplasm bound to the IκB inhibitory proteins. NF-κB-inducing stimuli activate the IκB kinase complex, IKKα and IKKβ, with the regulatory IKKγ/NEMO, resulting in sequential phosphorylation, ubiquitination, and degradation of IκB. Upon the detachment of IκB, the exposure of DNA-binding domain and nuclear localization sequence allows the NF-κB dimer to translocate to the nucleus to bind the target gene promoter regions (Karin et al., 2004; Meffert and Baltimore, 2005).

A body of evidence has shown that NF-κB plays a relevant role in regulating the function of immune system by driving both the diverse inflammatory phases and the host defense (Li and Verma, 2002).

In neurons, the transcription of both p50/RelA and p50/p50 NF-κB dimers can be activated by glutamatergic synaptic inputs through Ca^2+^/calmodulin-dependent protein kinase II (CaMKII) and local submembranous Ca^2+^ increase (Meffert et al., 2003). While the p50/p50 dimers localize in the cytosol, the p50/RelA dimers are found within synaptic boutons from where, upon glutamate or *N*-methyl-D-aspartate (NMDA) stimulation, they translocate to reach the nucleus and translate synaptic signals into altered gene expression (Meffert et al., 2003). Consistently, RelA knockout (ko) mice exhibit spatial learning deficits, thus supporting that NF-κB nuclear translocation and gene activation govern long-term changes to adult neuronal function caused by synaptic stimulation.

NF-κB controls adult neurogenesis in CNS (Rolls et al., 2007; Koo et al., 2010), and evidence supporting that these transcriptional regulators are important for ensuring Schwann cell differentiation and myelination of peripheral axons suggests that NF-κB factors are essential differentiation signals with a prominent role also in PNS development and plasticity (Nickols et al., 2003; Tang et al., 2013).

It has been found that most of the stimuli that activate NF-κB in the immune system, such as tumor necrosis factor-α (TNF-α) or interleukin 1 (IL-1), viral infections, and oxidative stress, exert the same effect in the CNS. Though surprisingly, TNF-α-mediated NF-κB activation plays a unique role in mediating neuronal plasticity in the hippocampus without inducing neuroinflammatory changes (Albensi and Mattson, 2000; Beattie et al., 2002). The NF-κB target genes in the CNS are only partially characterized, but it is predictable that these may display significant differences in their promoter organization when compared to the canonical genes affected in the immune system. On this line, we previously showed the presence of two NF-κB sites within the regulatory region of the DA D2 receptor (Bontempi et al., 2007) as a proof of the NF-κB involvement in the regulation of neuronal responses to DA-mediated transmission.

In glial cells, basal NF-κB activity is very low. For this reason, most studies have focused glial NF-κB in models of inflammation, injury, or disease (Dresselhaus and Meffert, 2019) where it is activated in its predominant form, the p50/RelA dimer (Kiebala et al., 2010; Simmons et al., 2016; Gupta et al., 2019). In inflammation, microglia activation results in the transcription of NF-κB-target genes, nitric oxide, IL-1β, and TNFα that in turn induce NF-κB signaling with consequent enhancement of inflammatory mediators that exacerbate neuronal cell death. NF-κB pathway in microglia seems also to actively participate in plasticity mechanisms and neuronal homeostasis in response to injury (Dresselhaus and Meffert, 2019). Likewise, beside inducing pro-inflammatory gene expression, astrocytic NF-κB appears to play a role in the central control of metabolism (Zhang et al., 2017) and, by promoting the clearing of glutamate from the synapses, in the termination of excitatory signals (Ghosh et al., 2011).

NF-κB has been reported to be essential for systemic and brain aging (Adler et al., 2008; Zhang et al., 2013), with RelA subunit mediating the most significant contribution to degenerative changes associated with senescence (Tilstra et al., 2012). On this line, NF-κB dysregulation has been found to participate in brain neurodegenerative mechanisms occurring in PD (Hunot et al., 1997), Alzheimer’s disease (AD) (Kaltschmidt et al., 1997; Chen et al., 2012; Jones and Kounatidis, 2017), as well as in post-traumatic or post-ischemic brain injury (Bethea et al., 1998; Schneider et al., 1999). With regard to the regulation of neuronal cell death, diverse NF-κB dimers in response to specific stimuli can mediate distinct responses (Pizzi et al., 2005; Lanzillotta et al., 2015). We showed that the selective inhibition of RelA or c-Rel expression produces opposite effects on neuron survival. Whereas the over-activation of p50/RelA dimers promotes apoptosis, activation of c-Rel-containing dimers improves the resilience of neuronal cells after injury (Pizzi et al., 2002, 2005; Sarnico et al., 2009b). Neurotoxic stimuli, such as ischemia, high glutamate concentrations, β-amyloid, or MPP+, induce the activation of p50/RelA dimers improving the transcription of proapoptotic genes (Pizzi et al., 2002, 2005; Inta et al., 2006; Valerio et al., 2006; Sarnico et al., 2008, 2009b; Yang et al., 2010). Conversely, c-Rel-containing dimers favor the expression of anti-apoptotic genes by signals promoting neuroprotection in diverse cell-based neurotoxic settings, such as IL-1β in NMDA-mediated excitotoxicity, mGlu5 receptor agonists in β-amyloid- or MPP+-mediated toxicity and adipocyte-derived hormone leptin in oxygen and glucose deprivation (OGD)-mediated apoptosis (Pizzi et al., 2002, 2005; Kogel et al., 2004; Valerio et al., 2006; Sarnico et al., 2008). Furthermore, the over-expression of c-Rel in cultured neurons promotes anti-apoptotic effects by inducing manganese superoxide dismutase (MnSOD) and Bcl-xL (Bernard et al., 2001; Pizzi et al., 2005). Overabundance of c-Rel also limits the generation of reactive oxygen species (ROS) by inducing transcription of the mitochondrial uncoupling proteins 4 (UCP4) (Ho et al., 2012), a brain-specific mitochondrial ion channel producing mild reduction in mitochondrial membrane potential and neuroprotection (Echtay, 2007).

The dual effects of NF-κB activation on neuron survival were corroborated by studies in severe brain ischemia models. Indeed, a rapid activation of p50/RelA in both neurons and glial cells has been implicated in the pathogenesis of post-ischemic injury (Herrmann et al., 2005; Crack et al., 2006). In ischemic brain tissue of mice subjected to permanent middle cerebral artery occlusion (MCAO) and in primary cortical neurons exposed to OGD, NF-κB followed a similar pattern of activation (Pizzi et al., 2009; Lanzillotta et al., 2010) characterized by increased nuclear translocation of p50/RelA dimers (Inta et al., 2006; Pizzi et al., 2009) and decreased translocation of c-Rel-containing dimers (Sarnico et al., 2009b). In these conditions, NF-κB activity was associated with an unbalanced expression of pro-apoptotic RelA target genes, with increased expression of the pro-apoptotic members of Bcl-2 family genes, Bim and Noxa, and parallel reduction of the anti-apoptotic member Bcl-xL (Cao et al., 2002; Inta et al., 2006; Sarnico et al., 2009b). During brain ischemia, RelA induced the expression of the 1B isoform of divalent metal transporter-1 (1B/DMT1), the membrane carrier responsible for iron accumulation and brain damage after injury (Ingrassia et al., 2012). This acted as an upstream mechanism responsible for iron accumulation and contributing to neuronal cell death. Knocking-down c-Rel expression exacerbated neuronal susceptibility to OGD-mediated damage and c-Rel ko mice exposed to cerebral ischemia resulted insensitive to the neuroprotective activity of leptin, a c-Rel inducer capable of limiting cortical damage in wt mice (Valerio et al., 2006, 2009). These data strongly suggested that inhibition of c-Rel-containing dimers and activation of p50/RelA are key events in the pathogenesis of post-ischemic brain injury.

Despite these premises, p50/RelA activation *per se* appeared to be insufficient to drive pro-apoptotic transcription during brain ischemia. Site-specific acetylation of RelA at the Lys 310 residue was necessary to switch anti-apoptotic p50/RelA, activated after a brief preconditioning ischemia and leading to brain tolerance toward the pro-apoptotic p50/RelA activated after a prolonged harmful brain ischemia (Blondeau et al., 2001; Lanzillotta et al., 2010). In this regard, studies showing that post-stroke induction of α-syn mediates ischemic brain damage (Ishimaru et al., 1998; Hu et al., 2006; Yoon et al., 2006; Unal-Cevik et al., 2011; Surgucheva et al., 2014; Kim T. et al., 2016) and that the levels of oligomeric form of α-syn of red blood cells in ischemic stroke and PD patients are both significantly higher than in controls (Zhao et al., 2016), seem to shed light on a possible link between NF-κB dysregulation and α-syn accumulation that deserves to be addressed by *ad hoc* studies.

## NF-κB Alterations in PD and Their Link to α-syn Pathology

A body of evidence has highlighted the occurrence of changes and dysregulation of NF-κB in PD. Post-mortem studies showed that in the brain of patients, there is an increased RelA nuclear translocation in melanized neurons of the *substantia nigra* that is supportive of NF-κB activation in PD (Hunot et al., 1997). Later studies, addressing glial involvement in the degeneration process of LB-bearing neurons on the post-mortem brain of patients affected by dementia with LB (DLB), found that some LB present NF-κB immunopositivity (Togo et al., 2001), thus hinting that NF-κB may participate in the α-syn-deposition-dependent neuronal loss. These findings were corroborated by other studies showing that either RelA or active phosphorylated NF-κB can be detected in the nucleus of subpopulations of neurons and glial cells of the *substantia nigra* of PD patients (Ghosh et al., 2007; Garcia-Esparcia et al., 2014). On this line, it has been described that α-syn can be internalized by microglia and induces nuclear accumulation of RelA (Cao et al., 2010, 2012). Moreover, Ghosh and co-authors found NF-κB activation in the *substantia nigra* of MPTP-treated mice. Mice treatment with a peptide corresponding to the NEMO-binding domain (NBD) of IKKα or IKKβ, which acts as a selective NF-κB inhibitor, reduced microglia activation in the *substantia nigra*, prevented both nigrostriatal axis degeneration, DA loss and improved motor functions (Ghosh et al., 2007). These findings confirmed that, by modulating microglia activation, NF-κB deregulation may play a role in MPTP mouse model. Consistently, Wang et al. (2020) have recently corroborated that c-Rel is rapidly upregulated in the *substantia nigra* and *striatum* of mice acutely exposed to MPTP treatment. They also found that c-Rel can maintain neuronal survival by promoting antiapoptotic gene expression in MPP+-exposed SH-SY5Y cells and inhibits LPS-induced BV2 cell activation by suppressing inflammatory gene expression, while the c-Rel inhibitor IT901 aggravated neuronal damage and microglia activation in the acute MPTP mouse model (Wang et al., 2020). Interestingly, they also reported a significant reduction in c-Rel expression in whole blood samples from PD patients (Wang et al., 2020), thus supporting that the loss of the protective role of c-Rel could play a role in PD-related neurodegeneration. It has also been shown that the expression of RING finger protein 11 (RNF11), a negative regulator of NF-κB signaling pathway localizing in LB (Anderson et al., 2007), is reduced in PD brains, and this feature correlates with increased phosphorylated form of activated RelA. Other studies support that NF-κB dysregulation may play a role in the control of α-syn expression (Liu et al., 2014). In particular, blockade of NF-κB signaling in a rat MPTP model was found to significantly decrease histone H3 acetylation in the α-syn SNCA promoter region, thus blunting α-syn in the *substantia nigra* and allowing recovery of the motor dysfunction (Liu et al., 2014). We recently described that mice deficient for c-Rel factor model most of the features of human PD (Baiguera et al., 2012; Parrella et al., 2019). In particular, at 18 months of age, they develop an L-Dopa-responsive parkinsonism, which associates with significant loss of nigral neurons and striatal fibers, reduction of DA levels, increased immunoreactivity for fibrillary α-syn, and cluster of differentiation 11b (CD11b)-positive microglia as well as iron accumulation with augmented DMT1 expression in the *substantia nigra* (Baiguera et al., 2012; Parrella et al., 2019). At 18 months of age, c-Rel ko mice also display a striatal increase in the proapoptotic form of RelA, carrying the site-specific acetylation at Lys 310 residue (Lanzillotta et al., 2015) that, in light of our previous findings (Sarnico et al., 2009b), supports the induction of neuronal damage. From 2 months of age, c-Rel-deficient mice suffer olfactory impairment and intestinal constipation associated with α-syn accumulation in the distal colon. At 5 months, they also start to exhibit progressive age-dependent deposition of fibrillary α-syn in the olfactory bulbs, *dorsal motor nucleus of vagus*, and *locus coeruleus.* The *substantia nigra* is affected by α-syn accumulation only from 12 months of age, when the striatal DA transporter drops, anticipating by 6 months the dopaminergic fiber degeneration. Finally, from 12 months onward, c-Rel ko mice exhibit oxidative/nitrosative stress in the *striatum* that parallels the altered expression of mitochondrial homeostasis regulators in the *substantia nigra* (Parrella et al., 2019). To assess whether inflammation and microglia activation accompany the onset and the progression of such PD-like pathology, we investigated the expression of cytokines and microglia/macrophage activation markers, together with microglial ionized calcium-binding adapter molecule 1 (Iba1) and astrocyte glial fibrillary acidic protein (GFAP) immunoreactivity, in the *substantia nigra* of c-Rel-deficient mice at both premotor and motor phase (Porrini et al., 2017). We observed increased expression of markers for alternative microglia/macrophage activation [mannose receptor C-type 1 (MRC 1) and arginase 1 (Arg1)] in 4-month-old c-Rel ko mice that, however, dropped in 13-month-old mice. At this age, they rather exhibited an increased expression of pro-inflammatory IL-1β, but not IL-6 or the microglia/macrophage phagocytic marker receptor for the Fc region of complexed immunoglobulins gamma (Fcgr3)/CD16, when compared to wt. At 18 months, c-Rel-deficient mice did not show significant variations in the transcription of inflammatory and microglia/macrophage activation genes when compared to age-matched wt littermates. Immunofluorescence analysis of Iba1-positive and GFAP-positive cells in the *substantia nigra* revealed no morphological changes in microglia and astrocytes in c-Rel ko mice along aging, while MRC1-Iba1-positive cells were identified as non-parenchymal macrophages only in 4-month-old animals. These observations indicate that c-Rel-deficient mice exhibit a mild brain inflammatory profile at a premotor phase (12 months) without evident signs of gliosis. Unexpectedly, this finding supports that a PD-like pathology can develop also in the absence of concomitant severe brain inflammatory process.

Taken together, our studies on c-Rel ko mice offer new insights into the pathogenesis of PD. They support that NF-κB unbalance increases the susceptibility of neurons to aging by promoting pathological changes, such as accumulation of α-syn and nigral neuronal loss, which are peculiar features of frail aged subjects over and above PD patients’ brain (Buchman et al., 2013, 2014). Indeed, the LB pathology incidentally detected in brain of people dying from non-neurological causes support that PD is an exacerbated version of aging, and if subjects could live longer, they would all develop the disease (Hawkes, 2008; Reeve et al., 2014). Therefore, alterations in c-Rel function may predispose people to PD development. What we miss are the mechanistic insights linking this phenomenon to α-syn accumulation and neurodegeneration. This notwithstanding, multiple pathways could be responsible for the onset of synucleinopathy in the brain of c-Rel ko mice. The peculiar energy-demanding profile of dopaminergic neurons, which depends on their distinctive morphological/structural and physiological features, predisposes them to mitochondrial shortfalls (Surmeier, 2018). The energy production by mitochondria and ER in the dopaminergic neurons of the *substantia nigra* associates with the generation of large amounts of ROS (Pacelli et al., 2015). ROS can be constantly neutralized by anti-oxidant systems, including superoxide dismutases (SODs), catalases, glutathione peroxidase, and UCP4 and UCP5. Remarkably, UCP4 and MnSOD are known to mediate the protective effects of c-Rel (Chen et al., 2000; Ho et al., 2012), supporting that, by blunting the expression of these genes, the absence of c-Rel might enhance ROS accumulation during aging in *substantia nigra* neurons. This process can synergize with reduced c-Rel-dependent expression of anti-apoptotic Bcl-xL (Sarnico et al., 2009a) to affect neuronal resilience. It is also conceivable that mitochondria impairment associated with c-Rel deficiency may first switch the acetylation state of RelA during aging to elevate Bim, DMT1, iron, as well as α-syn intracellular levels (Halliwell, 2006; Surmeier, 2018), which result into deposition of α-syn aggregates and neuronal damage.

It is worth mentioning that NFκB appears as a central and major regulator of protein aggregate clearance by modulating autophagic activity. Misincorporation of amino acid analogs into proteins as well as inhibition of proteasomal activity or expression of mutated SOD1 can induce a non-canonical NFκB activation that, in turn, upregulates the expression of two activators of selective autophagy, Bcl-2-associated athanogene 3 (BAG3), and heat shock protein B8 (HspB8), thus allowing clearance of protein aggregates (Nivon et al., 2016). Therefore, we may advance the hypothesis that a dysregulation of NF-κB deriving from c-Rel deficiency may predispose cells to misfolded protein accumulation, an effect that in the context of aging and PD may produce enhancement of α-syn accumulation as a main fall-out.

In addition to these possible explanations to justify how c-Rel deficiency may lead to PD, relevant clues may also derive from the fact that this factor is a key regulator of immunity by affecting T-cell differentiation and regulatory T-cell (Treg) function (Hilliard et al., 2002; Visekruna et al., 2012; Grinberg-Bleyer et al., 2017; Luu et al., 2017). Either innate or adaptive immune responses are implicated in PD (Kannarkat et al., 2013; Sulzer et al., 2017; Kustrimovic et al., 2019; Li et al., 2019; Tansey and Romero-Ramos, 2019). PD patients exhibit both brain and peripheral inflammation, and it has been found that inflammatory bowel disease predisposes to PD (Brudek, 2019). Notably, neuroinflammation promotes α-syn prion-like behavior, and along with aging, both the gastrointestinal tract and olfactory epithelium, which have been proposed as the initiation sites for α-syn spreading in the prion hypothesis, are mostly vulnerable to inflammation (Lema Tome et al., 2013). A body of evidence shows that by secreting antimicrobial peptides such as mucin or defensins or sensing pathogens via Toll-like receptors, enterocytes or intestinal epithelial cells represent the first barrier against gut microbiota (Magrone and Jirillo, 2013; Nagpal et al., 2018). Intestinal microbiota and gut immune system interact with each other, maintaining a condition of homeostasis in the context of the intestinal habitat. In daily interplay between normal microbiota (Balfour Sartor, 1997) and innate and adaptive immune cells, the more harmful bacteria species induce the release of pro-inflammatory IL-17 by T helper 17 (Th17) cells, which lead to IL-8 production by intestinal epithelial cells (Wu et al., 2009). Conversely, protective bacteria stimulate anti-inflammatory IL-10 by Treg cells that, by counteracting the activity of Th17 cells, avoid the noxious reaction to the host (Magrone and Jirillo, 2013).

As c-Rel is a crucial determinant of Treg identity and function (Grinberg-Bleyer et al., 2017), it may be easily conceivable that compromised Treg-mediated activity make c-Rel-deficient mice more vulnerable to microbiota-dependent bowel inflammation. The enteric inflammatory microenvironment, together with LPS, present at the outer membrane of Gram-negative bacteria, would, in turn, promote α-syn conformational shifts, aggregation, and spreading (Kim C. et al., 2016; Fitzgerald et al., 2019). Our observations, showing that young c-Rel ko mice exhibit α-syn accumulation in the distal colon (Parrella et al., 2019) anticipating the progressive α-syn brain deposition, sound in line with this hypothesis. Studies on patients with early stage diagnosed PD, showing that α-syn staining in the enteric nervous system correlates with compromised intestinal barrier integrity (Lema Tome et al., 2013) seem to further corroborate the above postulate.

## Conclusion

**FIGURE 1:**
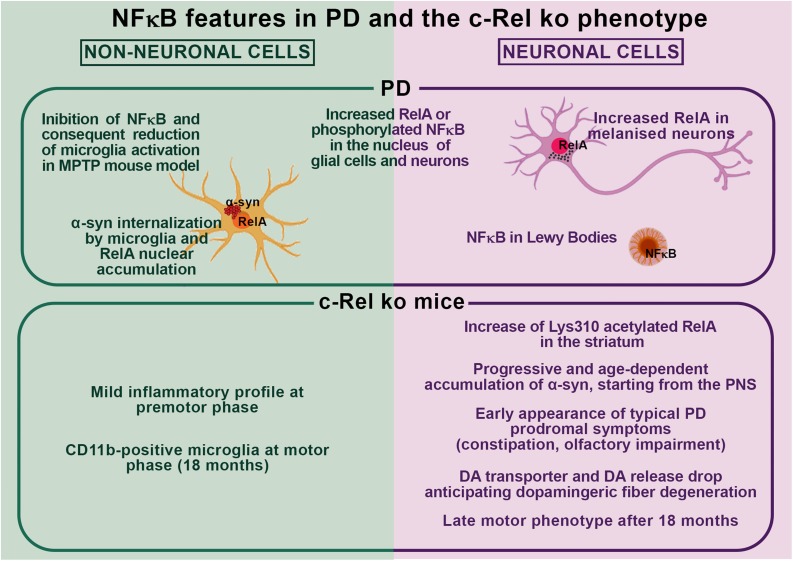
Schematic overview of nuclear factor-κB (NF-κB) features in Parkinson’s disease (PD) and in the c-Rel ko mouse model.

Collectively, these findings support the occurrence of a possible interplay between NF-κB dysregulation and α-syn pathology in PD as observed in c-Rel ko mice (Figure 1). In particular, by affecting key molecular pathways driving neuronal resilience to stressors, neuroinflammation, and protein misfolding, unbalances in the activation of NF-κB factors may significantly contribute to PD pathogenesis by favoring α-syn accumulation, aggregation and spreading, and promoting glial cell activation and neuronal cell death. These same processes may boost NF-κB activation, initiating a vicious circle perpetrating disease progression. Further studies are compelling to elucidate the causes and features of NF-κB alterations as well as whether and how NF-κB unbalance may lead to α-syn pathology and neurodegeneration in PD.

## Author Contributions

AB wrote the first draft of the manuscript. AB, LB, FL, GF, CM, EP, FB, VP, and MP revised the manuscript. AB, FL, FB, and EP prepared the figure and integrated it in the manuscript.

## Conflict of Interest

The authors declare that the research was conducted in the absence of any commercial or financial relationships that could be construed as a potential conflict of interest.
